# Panakeia - a universal tool for bacterial pangenome analysis

**DOI:** 10.1186/s12864-022-08303-3

**Published:** 2022-04-05

**Authors:** Sina Beier, Nicholas R Thomson

**Affiliations:** Parasites and Microbes, Wellcome Sanger Institute, Wellcome Genome Campus, Sulston Building, Hinxton, CB10 1RQ UK

**Keywords:** Bacteria, Whole genome sequencing, Pangenome

## Abstract

**Background:**

Development of new pan-genome analysis tools is important, as the pangenome of a microbial species has become an important method to define the diversity of a selected taxon, most commonly a species, in the last years. This enables comparison of strains from different ecological niches and can be used to define the functional potential in a bacterial population. It gives us a much better view of microbial genomics than can be gained from singular genomes which after all are just single representatives of a much more varied population.

**Results:**

We present Panakeia, a tool which strives to be easy to use and providing a detailed view of the pangenome structure which can efficiently be utilised for discovery, or further in-depth analysis, of features of interest. It analyses synteny and multiple structural patterns of the pangenome, giving insights into the biological diversity and evolution of the studied taxon. Panakeia hence provides both broad and detailed information on the structure of a pangenome, for diverse and highly clonal populations of bacteria.

**Conclusions:**

Previously published pangenome tools often reduce the information to a presence/absence matrix of unconnected genes or generate massive hard to interpret output graphs. However, Panakeia includes synteny and structural information and presents it in a way that can readily be used for further analysis. Panakeia can be downloaded at https://github.com/BioSina/Panakeiatogether with a detailed User Guide.

## Background

Pangenome analysis is increasingly popular, especially in microbiology, where the concept of species can be blurry at best [1, 2] and isolated single genomes are of limited value for understanding evolution and population diversity of microbes. With this interest in microbial populations comes the need to understand the complete genetic makeup from the individual isolates to the whole genus and more. The accumulated genome of these groups of samples is called a pangenome [3].

Several new tools have emerged in the last years to provide automated analysis pipelines. Each of them has a different focus into which functions of the pangenome are to be studied. Many investigate the size and members of common for the taxon compared to rarely found genes, generally called the core and accessory pangenome. In the last years, including analysis of syntenic structures in the pangenome has risen in importance, as these structures often include interesting functional operons, or provide important contextual information e.g. evidence of having been horizontally acquired. However, in large pangenomes, there can be many such loci or structures, and this makes it hard to determine which are essential and relevant for further investigation. Often, this information is presented in huge pangenome graph structures. These get increasingly complicated with growing number of input genomes and the inherent diversity in these genomes. These complexities can make it time-consuming and tedious for the user to interpret the results from pangenome analysis.

Here we describe Panakeia, an analysis pipeline for prokaryotic pangenomes which includes a graphic representation of the pangenomewith a special focus on analyzing the synteny and specific genomic patterns found in the dataset. Hence, Panakeia enables a detailed look into the structure of a pangenome, compared to the overview look of core and accessory genes that pangenome analysis tools generally provide. The syntenic structures Panakeia identifies can be filtered and divided into groups which hint at their function and origin using the provided post-processing scripts. The graph structure also inherently offers multiple ways to detect interesting structural patterns.

Panakeia is designed to be used together with the Pantagruel [4] pipeline for the reconstruction of the evolutionary history of all genes in the dataset. Pantagruel uses phylogenetic algorithms to determine the evolutionary history of genes and gene families. It can differentiate between evolutionary events and horizontal gene transfer and indicate if a gene transfer event has occurred between different lineages of the same species.

Together, the pangenome structure, including the synteny and patterns identifying small variants as well as large structural variants between the genomes determined by Panakeia and the genomic history of the genes determined by Pantagruel gives a detailed view of the evolutionary history and genomic plasticity of a set of closely related prokaryotic genomes.

## Implementation

Panakeia is written in Python 3, utilising the NetworkX [5] package for graph generation and analysis. It is split into a preprocessing script for clustering, the main pipeline and multiple post-processing scripts. We will now provide an overview on the implementation, a detailed user guide can be found on the Panakeia GitHub pages.

### Preprocessing

Input genomic DNA sequences for Panakeia should be annotated in GFF3 format, which is a standard output format for many prokaryotic annotation tools like Prokka [12] or PGAP [6]. For the following clustering step, the predicted protein sequences have to be available, either through the output of the annotation or by extracting them from the annotated genome using other tools. If annotation of the genomes is not feasible, Panakeia can be run without functional annotation of the genomes but predicted protein sequences have to be provided. They can be determined using fast protein prediction methods like Prodigal [7].

To analyse the pangenome, the proteins have to be clustered using the provided script *Clustering.py*, which takes a single fastA file with all predicted protein sequences from the genomes and the number of analysed strains (full genomes, not including plasimds or additional chromosomes as a separate count) as input. All proteins from the annotated genomes are clustered using cd-hit [8] in an iterative process, going from 90% sequence similarity required to cluster proteins over 80% similarity and 75% down to 70%. Clusters which include at least the number of input genomes are kept in each step, while proteins in smaller clusters are re-clustered with the next lower similarity threshold until the threshold of 70% sequence similarity is reached. At this point, all remaining clusters are kept. One random sequence is chosen as a representative for each cluster.

### Pan-genome analysis

The main analysis step is done by the *Panakeia.py* script. Panakeia reads in the genome annotations for each input genome in GFF3 format, generating so-called strain graphs, which are graphs using protein clusters as nodes and connecting local neighbours with edges. Local neighbors are defined as protein clusters from genes which are direct successors or predecessors of each other on the annotated sequence. Another type of edge is added for paralogs, which are detected by reading in the clusters generated by the previous step and defining each pair of proteins which are from the same genome and classified into the same cluster as paralogs.

A strain graph is generated for each input genome (each separate GFF3 file), which means they will include both the chromosomal and any plasmid sequence found in a genome. We define these input genomes as strains, as generally in bacterial pangenome analysis they would be strains of the analysed species.

The clusters are also used as nodes for a pangenome graph, which is similar to the strain graphs but has whole clusters as nodes (annotated by the features of the clusters representative sequence). Edges are added to the pangenome graph if two clusters include neighbouring proteins in at least one of the input genomes. Thus the edges determine the synteny information for the pangenome graph. Edge weight is determined by the number of genomes in which the connected nodes are neighbouring each other. Pangenome nodes also include information on the maximum number of paralogs predicted proteins from the cluster can have in one single genome.

These paralogous clusters are then subdivided into subclusters by determining the minimal number of unrelated neighbourhoods the cluster is part of in all genomes. Each neighbourhood generates a subcluster, with the original cluster nodes being removed from the graph. Subcluster nodes share the same representative, but the features determining in which strains they occur and if they belong to the pangenome core or accessory proteome are updated for each subcluster.

All protein clusters from the dataset will be part of the pangenome graph, but the addition of edges can be influenced by setting a parameter to require a minimum weight for the edge to be added. This can help to remove clutter generated by assembly errors in singular input genomes and is especially helpful when analysing large datasets.

### Associating protein clusters with chromosomal information

If finished or nearly finished genomes which are assembled in the correct number of expected contigs (chromosomes or plasmids) are available, the pangenome graph can be annotated with basic structural information using the *ChromosomizeAll.py* and *ChromosomizePangenome.py* scripts. We will refer to these finished genomes as template genomes. Clusters which have a member found in one of template genomes are assigned to a chromosome, undetermined (if they were present on either chromosome/plasmid) and unknown (if the cluster was not present in any of the complete assemblies). Chromosomal information is added to the protein clusters in the pangenome graph and all proteins (determined by their cluster membership) in the strain graphs using the information from the template genomes. This process will further be called ’chromosomizing’ the genome.

### Determining structural patterns in the pangenome

Panakeia can detect different types of patterns in the pangenome graph using the *Patterns.py* script, which potentially correspond with specific biological features. The detected patterns are

- orphans
- uniques
- variants
- insertions
- indels

The implied meaning of these patterns is further described in the results.

Patterns are detected from the pangenome graph by utilizing graph algorithms. The detected patterns are returned as text-based files - either lists or tables, depending on the type of pattern - and as separate graph files which includes only the occurrences of each pattern. This makes it possible to either look at them separately, or overlay the pattern information onto the full pangenome graph by using the *HighlightSubgraph.py* script which uses one of the pattern graphs to highlight the pangenome graph.

### Highlighting external information

If phylogenetic information or other information clustering or grouping the input genomes - for example lineages defined by Pantagruel or groups defined through metadata features - are available to the user, groups of genomes can be highlighted onto the pangenome graph by using the *HighlightStrains.py* script, which creates features in the pangenome graph to highlight protein clusters and local neighborhood relations found in a strain through visualisation. This will highlight protein clusters and neighborhood relations occurring in a list of strains. Protein clusters and edges already included in the pangenome graph will be marked in orange. Protein clusters and edges which did not reach the threshold for minimal weight in the pangenome graph will be added and highlighted in yellow. This enables the user to depict the part of the pangenome and functional potential covered by a defined group of genomes and also extract a group-specific sub-pangenome more easily using network visualisation tools.

### Inclusion of pantagruel output

Panakeia is designed to work closely together with the Pantagruel [4] pipeline for reconstruction of gene histories in bacterial pangenome datasets. Pantagruel output includes information on phylogenetic clades for the input genomes and genes specifically present in these clades or specifically absent compared to neighbouring clades. This presence and absence information informs about evolutionary changes of gene uptake or loss in defined phylogenetic clades which might be related to the fitness in a specific environmental niche or the virulence of pathogenic genomes. The *HighlightClades.py* script uses the Pantagruel output files, specifically the species tree clade definition file and a version of a clade-specific gene set file including either the specifically present or absent genes, to highlight all protein clusters including proteins from the genomes in the clade and mark either the specifically present or absent protein clusters. This highlighting connects the synteny information to the evolutionary history of these groups of genes.

## Results

### Running pankeia

To prepare for running Panakeia, the user will need the protein sequences extracted from annotation or protein prediction for all the input genomes in a single multi-fastA file, as well as the matching GFF3 files with protein annotations for each separate genome. To produce the protein clusters, the protein fastA will be used as input for clustering the protein sequences with the provided clustering script. This script returns a file containing all cluster information and another file with all the representative sequences for each cluster, including all the functional annotations, if they have been provided in the input GFFs. The main Panakeia pipeline then requires selection of an output directory for all results, an input directory which has to include all GFF3 files from the input genomes, the clustering output file and the file with the representative sequences as they are provided by *Clustering.py*.

Additionally, parameters can be provided defining the percentages required to identify a protein as belonging to the different partitions which compromise the pangenome: hard core, soft core and shell. Hard core represents protein clusters which strongly belong to the backbone of the investigated taxon and should not be missing from any genomes. Soft core protein clusters occur in most input genomes, but are not essential to the taxon, so might be missing occasionally. Shell protein clusters occur in groups of genomes, and potentially include the structure which differentiate between lineages or functional groups of organisms. Finally, the left over protein clusters belong to the cloud, which includes only proteins which occur rarely or even in singular genomes. The cloud often includes erroneous protein predictions caused by assembly errors, but also new horizontal gene transfers.

The default values for these parameters are set as 0.99 (meaning a protein has to occur in 99% of the genomes to be counted, acoounting for some proteins missing due to sequencing and assembly errors) for hard core, 0.95 to count it into soft core and 0.15 to count it to the shell. Everything occurring in less than 15% of the genomes will, in this case, be counted as belonging to the cloud. It is also possible to set a parameter defining the minimal number of occurrences in the pangenome for an edge (local neighbourhood connection between to proteins) to be drawn. This defaults to drawing all edges. For large numbers of input genomes it can be helpful to set this parameter to remove edges which only occur in single genomes or very small numbers of genomes. This automatically reduces the prevalence of assembly errors and generates aq more simplified version of the pangenome graph.

### Panakeia output

#### Genome graphs

**Fig. 1:**
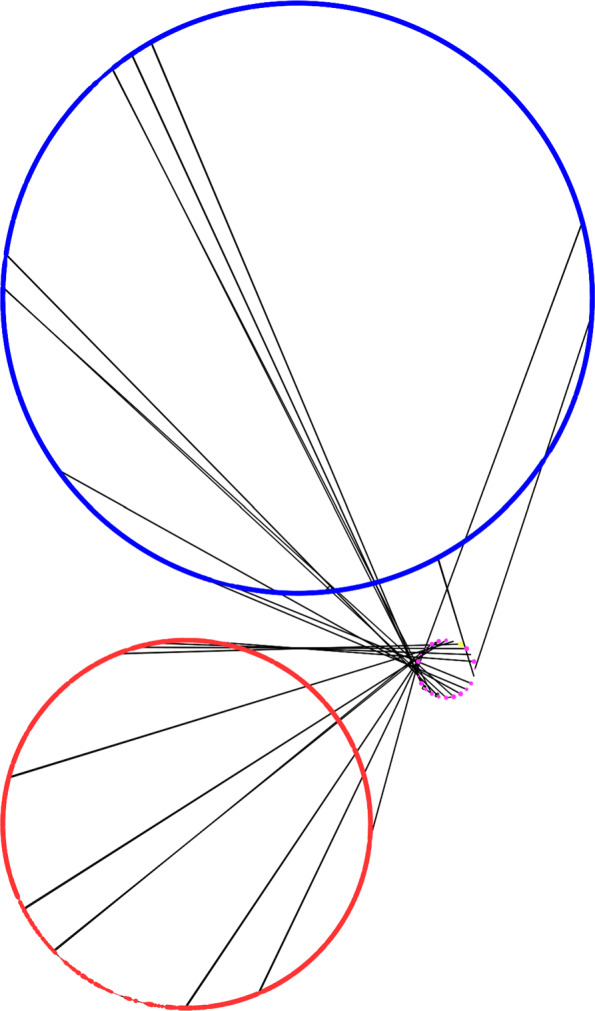
Chromosomised view of a strain graph for a *V. cholerae* strain M66 genome. *Vibrio* species have two chromosomes, which are clearly shown as the two large circles. Proteins found only on chromosome 1 in the are shown as blue nodes, proteins uniquely found on chromosome 2 are shown as red nodes, proteins, where the chromosome cannot be decided as they occur on both chromosomes in the template genomes, are shown as pink nodes in the smallest circle and proteins which cannot be assigned a chromosome as they do not occur in the template genomes would be shown as grey nodes. The protein nodes are connected to their local neighbours on the contigs though black edges. Contigs from the input genome are hence denoted by nodes connected through black edges and it the chosen attribute based visualisation from Cytoscape [9] clearly shows the two chromosomes as circles and a separate small circle including only the proteins which cannot be placed

**Fig. 2:**
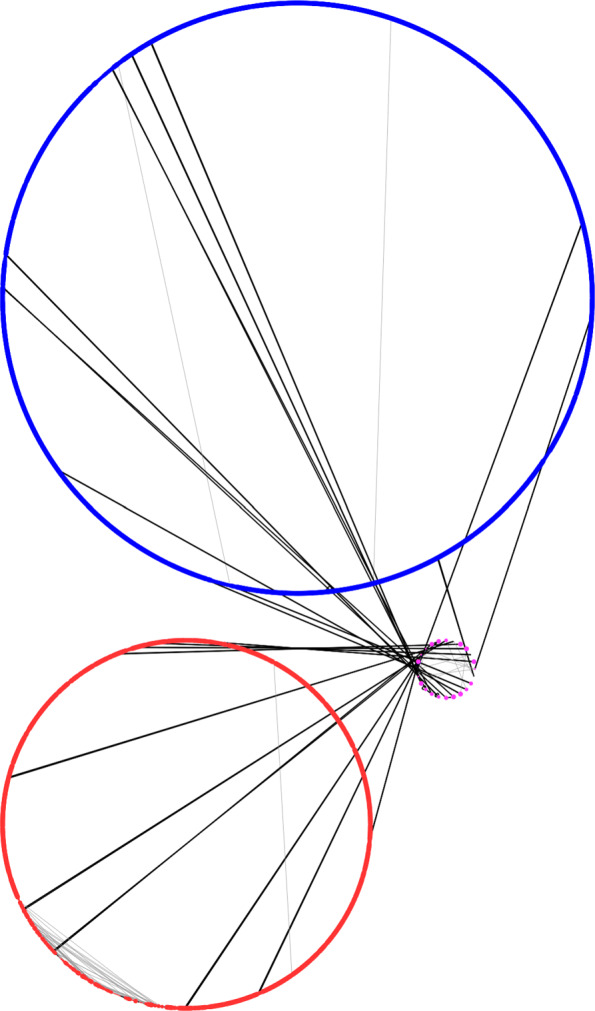
Chromosomised view of a strain graph for a *V. cholerae* strain M66 genome. *Vibrio* species have two chromosomes, which are clearly shown as the two large circles. Proteins found only on chromosome 1 in the are shown as blue nodes, proteins uniquely found on chromosome 2 are shown as red nodes, proteins, where the chromosome cannot be decided as they occur on both chromosomes in the template genomes, are shown as pink nodes in the smallest circle and proteins which cannot be assigned a chromosome as they do not occur in the template genomes would be shown as grey nodes. The protein nodes are connected to their local neighbours on the contigs though black edges and paralogous proteins are connected to each other with grey edges. Contigs from the input genome are hence denoted by nodes connected through black edges and it the chosen attribute based visualisation from Cytoscape [9] clearly shows the two chromosomes as circles and a separate small circle including only the proteins which cannot be placed. In addition here a region including many paralogous proteins connected by grey edges in chromosome 2 can be seen on the lower left part of chromosome 2. This denotes the known integron island of *V. cholerae*, which introduces a high structural variability between strains and includes multiple paralogous proteins which in this view create the light grey edges to connect paralogs

Panakeia generates a so-called *strain graph* for each input genome, which is provided in the GraphML format that can be loaded into many commonly available graph visualisation tools, including Cytoscape [9]. The strain graphs can then be chromosomised of finished or high-quality reference genomes are available amongst the input genomes to help define the different chromosomes and/or plasmids. An example for a chromosomised version of a *Vibrio cholerae* genome from strain M66 (chromosome 1: NC_012578, chromosome 2: NC_012580) is provided in Figs. 1 and 2.

**Fig. 3:**
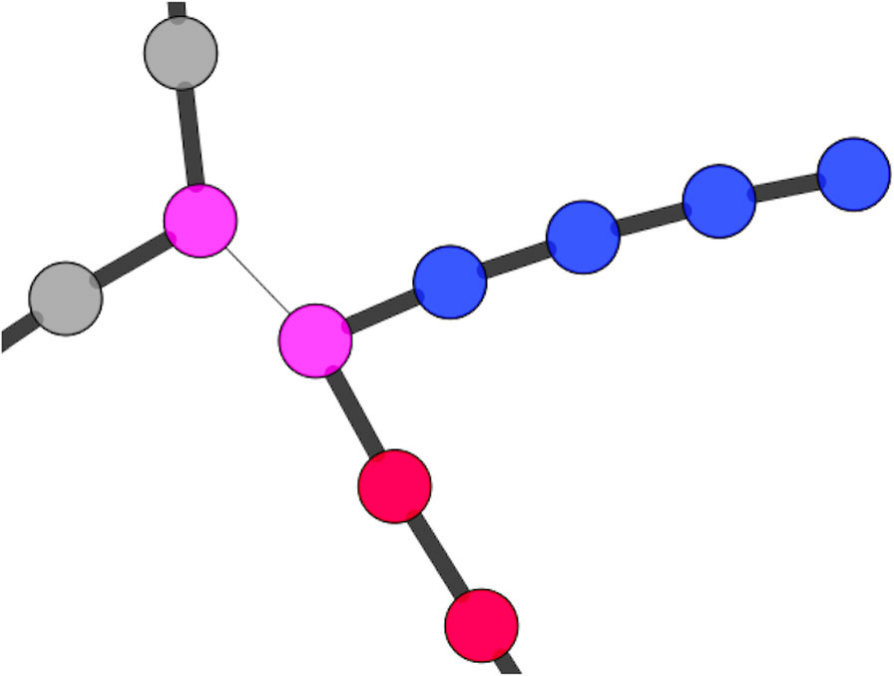
Zooming in on a chromosomized strain graph of a *V. cholerae* strain with two chromosomes shows a misassembly in the genome. Blue nodes represent proteins belonging to chromosome 1 and red nodes represent proteins belonging to chromosome two. The pink node represents a protein marked as undecided, because it has been found on either of the chromosomes in the template genome. Wide black edges mark local neighbourhood relation, the thin grey edge marks paralogous relation of two proteins. The undecided protein clusters are annotated as IS200/IS605 family transposases. The same transposase occuring multiple times in one genome might have lead to the misassembly because it creates a repeat region

Strain graphs help to assess the assembly of an isolate, as assembly errors caused by repetitive sequences often show up as loops of paralogous proteins in the synteny of the strain graph. Assembly errors caused by single paralogs or small mobile elements are easily found in chromosomised strain graphs as they have a change of chromosome/plasmid assignment around a single or a group of proteins which occur on both chromosomes, as shown in Fig. 3.

Strain graphs also serve as input for further processing like *HighlightStrains.py*, which can be used to highlight one or more strains as an overlay over the complete pangenome, and scripts specific to include output of the Pantagruel [4] pipeline into the pangenome graph.

#### Pangenome graph and analysis

**Fig. 4:**
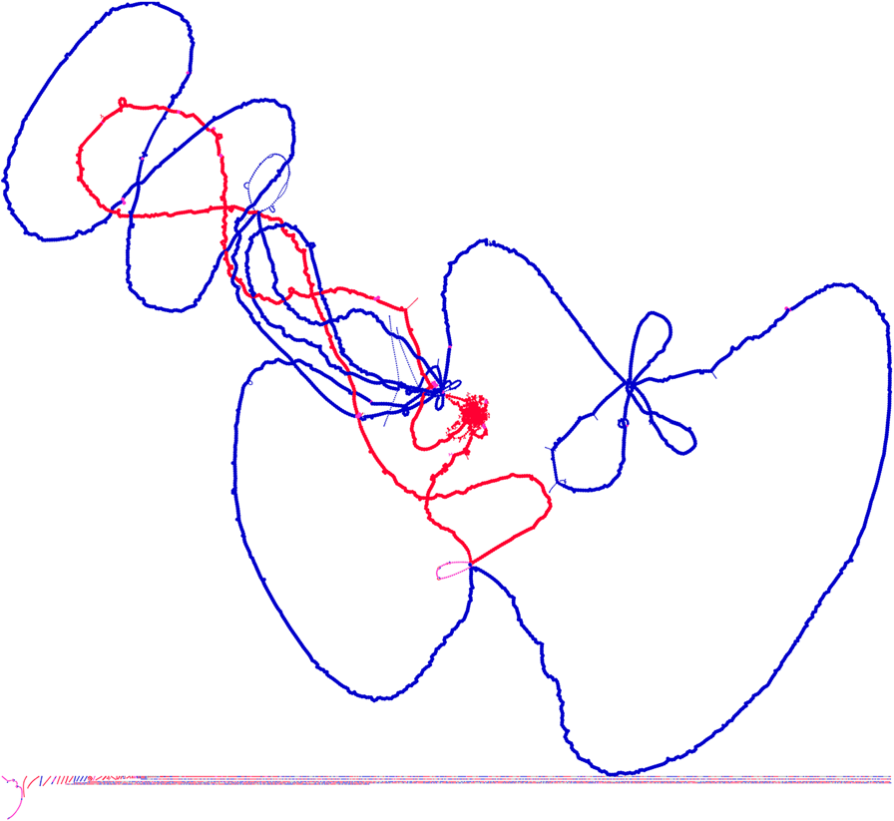
Chromosomised view of a pangenome graph for 42 finished *V. cholerae* as found on RefSeq. Nodes represent protein clusters, edges represent local neighborhood relations of proteins in the clusters. As the species has two chromosomes, protein clusters from chromosome 1 are colored blue, and protein clusters from chromosome 2 are colored red, protein clusters, where the chromosome cannot be decided as they occur on both chromosomes in the template genomes, are coloured pink and protein clusters which cannot be assigned a chromosome as they do not occur in the templates are colored grey. The size of the nodes is defined by how many strains the respective protein cluster includes, meaning core clusters are larger and protein clusters from the shell and cloud are smaller. We would expect two large circles from the two chromosomes, but they are connected through protein clusters including proteins on either chromosome. Loops represent structural rearrangements and inDel regions in parts of the pangenome. The smaller connected components unconnected to the main chromosomal components represent either rare insertions - potentially horizontal gene transfer - or variants, sequences created by contamination of some of the input genomes or assembly and annotation artefacts. The graph was generated by only allowing edges with a minimal support of 2 to be added, to single out these potentially erroneous protein clusters

The full pangenome graph is the most important output of the Panakeia pipeline, it enables all further analyses steps and visualisation of the data. This graph includes all protein clusters and all local edges for neighborhood connections that occur in more genomes than the given minimal support threshold. The nodes hold information like the strains they occur in if they are hard core, soft core, accessory or cloud, the representative protein of the cluster, the maximal number of paralogs in a genome, number of proteins in the cluster and a weighting based on the average number of proteins in this cluster per genome. If available, it also includes the functional annotation of the cluster, and if the pangenome has been chromosomised previously, it includes the chromosomal prediction. An example of a chromosomised pangenome graph is shown in Fig. 4.

#### Pattern extraction

After the pangenome graph is generated and annotated, biologically relevant patterns can be detected using the *Pattern.py* script, as described earlier in Implementation.

Orphans are protein clusters which do not have a common placement in the genomes. They are either caused by contigs from contamination of the genomic sample with external DNA, assembly problems around this specific gene or highly variable locations in different genomes. The latter can hint at the proteins belonging to small mobile elements. The functional annotation can help differentiate between these cases. Orphans are detected by selecting nodes in the graph without any connection to other nodes, but present in multiple genomes.

‘Uniques’ are protein clusters which only occur in a single genome. They are either caused by contamination or annotation errors or are truly novel, perhaps as a result of recent horizontal gene transfer into that genome. This is when combining information from Pantagruel can be especially useful because it can be used to further investigate their evolutionary history.

‘Variants’ are protein clusters which have multiple amino acid variants occuring in the same genomic neighborhood. This only occurs if the sequence variation is bigger than the cutoff used for clustering. They can be variants of the same functional gene with small changes in the amino acid sequence, but also include truncated proteins or pseudogenes which have lost their function in a part of the genomes.

To detect variants, small bubbles of multiple nodes are detected, which have a connection to nodes outside the bubble only on one or two of the nodes. Commonly these represent the existence of multiple variants of the same gene, with less than 70% aminoacid sequence similarity.

‘Insertions’ stand for rare short insertions into one or a few genomes. Much like the Uniques, they can be caused by contamination but also can hint at horizontal gene transfer or incorporation of a plasmid or other mobile genetic element. Insertions are small bubbles in the graph formed by insertion of one or more protein clusters which only involve clusters assigned to the shell or cloud of the pangenome.

‘InDels’ are common insertions or rare deletions (relative to the number of input genomes) that are found in the pangenome. These are potentially structural variants and hint at gene loss in a few of the genomes or at large-scale homologous recombination events, which can be typically seen around capsule or O-antigen gene clusters.

InDels are cycles in the graph which involve more than four nodes and are not restricted by how common any of the clusters occur in comparison to the insertions.

**Fig. 5:**
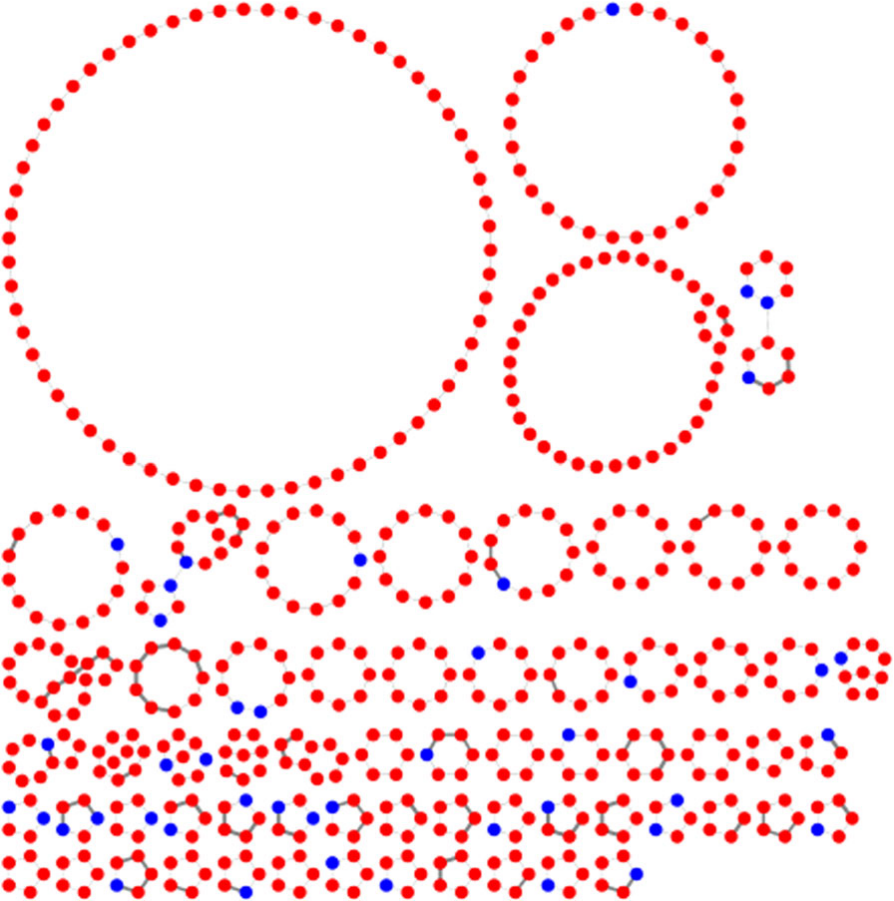
This Pangenome Subgraph is the result of pattern detection for InDels on a set of 318 genomes. Shown are only the protein clusters and edges belonging to any of the InDels detected by *Patterns.py*. The core (hard and soft) protein clusters are marked blue, the non-core (shell and cloud))protein clusters in red. InDels often belong to the shell (marking an insertion into a few strains). Very rare deletions are not detected by this analysis, as they would still be part of the core of the pangenome. InDels are often “attached” to the core through one or two core protein clusters which mark the location of the inDel. If they are attached to the hard core this way, inDels are either inherited in most genomes or represent a mobile element specific to a single insertion position

The *Pattern.py* script can also generate a tabular representation of the presence/absence matrix for all protein clusters and genomes in the dataset. Patterns extracted from the pangenome are saved either in tabular or text form, depending on the pattern. They are also saved as a graph only containing the occurrences of the pattern for visualization. An example of an extracted InDel graph is shown in Fig. 5. The matching text output includes a tab-delimited list of clusters for each InDel, displayed in one InDel per line. Text output for variants is similar, with a list of the varying different protein clusters in the same structural location per line.

Text output for insertion sequences is in tabular form, including the node degree (number of attached edges) and node weight for each potential insertion sequence.

### Comparison to other pangenome tools

Panakeia uses a simple approach to clustering proteins compared to other pangenome tools and focuses on using the synteny and paralogy information from the input genomes to increase the available information content and accuracy of the resulting pangenome graph. Synteny information is also used by over novel tools like Panaroo [10] and PPanGGOLiN [11], which do cluster genes instead of proteins. In general, pangenome tools often specialise by either in showing an overview of the full functional diversity of a studied taxon or by correcting small errors caused by technical or algorithmic restrictions and giving information on a long list of small detailed structures. Both approaches output can be cumbersome and need prior knowledge to interpret and utilise the information appropriately.

Panakeia incorporates no default correction steps other than splitting paralogous clusters and enabling the user to set a cutoff for minimum support of edges, but it can give specific insights by using the various patterns described in Implementation together with the functional annotation. This makes it possible to move from a broad view of the available diversity to details about interesting structures and patterns which define the difference between groups of genomes and can lead to helpful insights about the evolution and adaptation of these organisms.

To explain the differences of Panakeia to other pangenome tools, here we will compare it to two relatively current developments in the field and define the different use cases for each of the tools.

PPanGGOLiN [11] is named after the Partitioned PanGenome Graph Of Linked Neighbors it uses to analyse the pangenome. It focuses on partitioning the homologous gene clusters it uses into a persistent (core), cloud and one or more shell partitions. Panakeia in comparison divides the core into hard and soft core to be able to track novel gene loss which has only occurred in a small subset of the taxons phylogeny through using the pattern detection to detect InDels in the backbone of the pangenome. Panakeia does not further divide the shell - which is usually the largest group of protein clusters for large and diverse datasets - because these clusters are the most important to be able to detect the patterns associated with features of a group of genomes. Dividing the shell into further groups would potentially restrict the size of a group of genomes which include one of these patterns for them to be found. Gautreau *et al.* [11] mention the possibility of predicting genomic islands in the shell and cloud as a future option of PPaNGOLiN, Panakeia already includes the functionality through the pattern search.

Panaroo [10] focuses on correcting the inclusion of genes into the pangenome, filtering out potential contamination and genes classified as being based on misassembly or annotation errors. This leads to very clean pangenome graphs even for large input datasets but could result in the loss of new horizontal gene transfer events or changes in the structure caused by the movement of mobile elements. Hence this approach might not be appropriate for taxa with known or predicted high diversity, high genome plasticity or even just for datasets where more fragmented assemblies have to be included. By using the parameter to set a minimum weight for edges to be included in the pangenome graph, Panakeia can be set up to filter out many similar errors easily, as they are generally creating rare or even unique edges. The potential errors will show up as singletons in the pangenome graph and can then be detected by the pattern search. Many visualisation tools will also allow you to detect the singletons as nodes of degree 0 and remove them from the graph for a cleaner visualisation, making it unnecessary to already include this detection when building the pangenome graph.

## Discussion

Panakeia is designed to combine pan-genome analysis with genomic information from isolate genomes to improve the information collected both about the studied population and the genome plasticity and structure of single members in it. Additionally, it can be linked to phylogenetic and evolutionary information provided by Pantagruel. This combination will help to improve our understanding of how microbial genomes function, adapt and evolve. Automatically extracting and highlighting interesting patterns enables researchers to focus on potentially interesting features of the genomes without the need of painstakingly finding them in long lists, tables and huge graphs.

Based on Python 3 with minimal extra packages and standardised input and output file formats, the pipeline is widely applicable and easy to install, maintain and use.

The pipeline is not optimised for speed, but aimed at reducing the manual work necessary to find patterns of interest in large pangenome datasets, which in turn reduces the time needed to extract useful information from these datasets. The information about the patterns can be exported both in text-based files which are easy to use in further analysis and as GraphML files for visualisation and manual curation of the results. Hence, Panakeia is a tool which is usable in many settings and for people from different backgrounds and with different requirements for their work.

## Conclusions

With Panakeia, we provide a novel tool for pangenome analysis and visualisation. Available tools are often lacking either in the type of analysis by providing only basic statistics, lacking proper visualisation and interpretability of their results or providing little documentation and hence being not user-friendly. Panakeia overcomes these hurdles and is available as an open-source, free to use software without the need for powerful computational infrastructure or specialist bioinformatics knowledge.

## Availability and requirements

**Project name:** Panakeia

**Project home page:** https://github.com/BioSina/Panakeia

**Operating system(s):** Platform independent

**Programming language:** Python

**Other requirements:** Python 3.0 or higher

**License:** GPL 3.0

Open source, free for non-academic use

## Data Availability

Code, Cytoscape layout files and example datasets are available on the Panakeia GitHub page (https://github.com/BioSina/Panakeia).
